# Evaluation of the content validity index of the Australian/Canadian osteoarthritis hand index, the patient-rated wrist/hand evaluation and the thumb disability exam in people with hand arthritis

**DOI:** 10.1186/s12955-020-01556-0

**Published:** 2020-09-09

**Authors:** Pavlos Bobos, Joy C. MacDermid, Eleni C. Boutsikari, Emily A. Lalone, Louis Ferreira, Ruby Grewal

**Affiliations:** 1 School of Physical Therapy, Department of Health and Rehabilitation Sciences, Western University, London, ON Canada; 2 Western’s Bone and Joint Institute, Collaborative Program of Musculoskeletal Health Research, Western University, London, ON Canada; 3 Roth McFarlane Hand and Upper Limb Centre, St. Joseph’s Hospital, London, ON Canada; 4 Dalla Lana School of Public Health, Institute of Health Policy, Management and Evaluation, Department of Clinical Epidemiology and Health Care Research, University of Toronto, Toronto, ON Canada; 5 McMaster University, Hamilton, ON Canada; 6 Department of Hygiene, Epidemiology and Medical Statistics, National and Kapodistrian University of Athens, Athens, Greece; 7 Department of Mechanical and Materials Engineering, Western University, London, ON Canada

**Keywords:** Osteoarthritis, Rheumatoid arthritis, Psoriatic arthritis, Content validity, Hand arthritis

## Abstract

**Background:**

The Australian/Canadian Osteoarthritis Hand Index (AUSCAN), the Patient-Rated Wrist/Hand Evaluation (PRWHE) and the Thumb Disability Exam (TDX) are patient-reported outcome measures (PROM) designed to assess pain and hand function in patients with hand arthritis, hand pain and disability, or thumb pathology respectively. This study evaluated the content validity of AUSCAN, PRWHE and TDX in people with hand arthritis.

**Methods:**

This study enrolled participants with hand arthritis to rate the items of all 3 PROM in terms of relevance and clarity. The Content Validity Index (CVI) was computed for each item in each scale (I-CVI) as well as for the overall scale (S-CVI). Kappa was used to determine the inter-rater agreement among the raters.

**Results:**

Overall, 64 individuals with hand arthritis (27% with OA, 67% with rheumatoid arthritis and 6% with psoriatic arthritis) participated in the study. The I-CVI for all items and all scales were very high (I-CVI > 0.76) and the modified Kappa agreement among the raters demonstrated excellent agreement (k > 0.76). The S-CVI for all PROMs was very high for relevance (AUSCAN = 0.92, 95% CI 0.90 to 0.94; PRWHE = 0.85, 95% CI 0.82 to 0.88 and TDX = 0.87, 95% CI 0.85 to 0.89) and for clarity (AUSCAN = 0.99, 95% CI 0.98 to 1.00; PRWHE = 0.95, 95% CI 0.93 to 0.97 and TDX = 0.91, 95% CI 0.89 to 0.94), respectively.

**Conclusions:**

This study demonstrated very high content validity indices for the AUSCAN, PRWHE and TDX; with strong consensus across raters. This augments prior studies demonstrating appropriate statistical measurement properties, to provide confidence that all three measures assess important patient concepts of pain and disability.

## Introduction

Hand osteoarthritis (OA) is one of the most common musculoskeletal diseases and a leading cause of disability with an increasing prevalence mainly attributed to increased life expectancy [1, 2]. Clinical characteristics of hand OA typically involve pain, reduced hand function, decreased hand grip strength, poor quality of life [3, 4] joint degeneration, bony enlargements and joint swelling [5]. Rheumatoid arthritis, although leading to bone tissue abnormalities, loss of joint function and impact on quality of life similarly to OA, is a distinct pathology that mainly targets synovial and soft tissue structures [6].

Patient-reported outcome measures (PROMs) are often administered to assess any health-related changes that may have occurred as a consequence of health-management interventions [7, 8]. Many properties are important [9–13] during an instrument development such as reliability and validity but a key property is considered to be content validity [14]. Content validity can be defined as the degree of which the instrument or the questionnaire is an adequate reflection of the construct being measured [15]. Based on the Consensus-based Standards of the selection of health Measurement Instruments (COSMIN) initiative content validity is considered as one of the most important measurement properties [14]. While reliability, responsiveness and other types of validity can be pivotal for an outcome assessment they may be insufficient to establish the validity of a PROM [16]. When PROMs include irrelevant items and lack of clarity they are inefficient, and may have weaker measurement properties [14]. Most importantly, if key aspects are missing or the questions are not relevant responses, they may not reflect patient status or concerns, and may be biased because patients may get frustrated [17].

The Australian/Canadian Osteoarthritis Hand Index (AUSCAN) [18], the Patient-Rated Wrist/Hand Evaluation (PRWHE) [18] and the Thumb Disability Exam (TDX) [19] are clinical tools designed to assess pain and hand function in hand arthritis [18–21]. Both AUSCAN and PRWHE have demonstrated construct validity with verbal rating scale, had high internal consistency, and correlated with each other at baseline and follow-up time points in patients with early thumb carpometacarpal OA [18]. However, previous studies have reported inconsistent results about construct validity of AUSCAN [22–24]. Haugen et al. showed that AUSCAN total index lacks construct validity with items contributing to separate scales of pain, stiffness, and physical functioning [24]. Also, a recent update of PRWHE was performed to improve the clarity and applicability of items, but this version has not been compared to the AUSCAN and it is important to assess the content validity of the revised scale. The TDX is a more recently developed scale that has not been compared to either the PRWHE or AUSCAN. Although, previous studies have demonstrated appropriate statistical measurement properties, content validity evaluations are needed to ensure that the constructs being evaluated are those intended, and that items are interpreted probably by potential respondents. Limited investigation of content validity has been reported for any of these three questionnaires. Therefore, we aimed to investigate the quantification of content validity index by asking patients with hand arthritis to rate each of the instruments items in terms of relevance and clarity.

### Primary objective

To evaluate the Content Validity Index (CVI) of the Australian/Canadian Osteoarthritis Hand Index (AUSCAN), the Patient-Rated Wrist/Hand Evaluation (PRWHE), and the Thumb Disability Exam (TDX) in patients with hand arthritis.

## Methods

### Study design

This study was a cross-sectional design that investigated the content validity of patient-reported outcomes (AUSCAN, PRWHE and TDX) for hand arthritis. Ethical approval was granted from the Hamilton Integrated Research Ethics Board (HiREB).

#### Inclusion criteria

1. The participant was able and willing to provide informed consent
2. Participants were between 18-85 years old
3. The participant had hand arthritis.
4. The participant can read and write English.

#### Exclusion criteria

1. Hand pathologies or conditions other than arthritis
2. Inability to answer the survey questions in English.

### Setting and recruitment

Participants were recruited through poster advertisements at The Roth McFarlane Hand and Upper Limb Centre (HULC) at St. Joseph’s Health Care Hospital in London, Ontario and through The Arthritis Society main website. The patients that expressed interest to participate in the study received a letter of information about the survey. Both electronic and paper versions of the survey were available for participants. An email with the link of the online survey was sent out to the participants that were interested to complete the electronic version. The electronic version was hosted on Qualtrics from May 2019 till February 2020 which is a secure data collection platform [25]. Participants were asked to provide consent to proceed into the survey questions. Allthe items were rated for relevance and clarity in an order (AUSCAN, PRWHE, TDX). Participants were asked to rate the relevance and clarity of each item of AUSCAN, PRWHE and TDX.

### Patient-reported outcome measures

The Australian/Canadian Osteoarthritis Hand Index (AUSCAN) is a 15-item self-reported disease specific questionnaire measuring pain (5-items), function (9-items) and stiffness (1-item) in the hand on a scale from 0 – none to 4 – extreme for all items [18, 20]. The Patient-Rated Wrist/Hand Evaluation (PRWHE) is a self-administered questionnaire which has 2 subscales of pain (5-items) and function (10-items). The PRWHE was originally developed and tested for people with distal radius fracture (DRF) [21, 26, 27] and later validated as applicable to the wrist/hand for multiple conditions including arthritis as the PRWHE [18, 28]. Each item is scored from 0 to 10 scale which 10 indicates the worst possible pain or disability. The Thumb Disability Exam (TDX) is composed of 20 questions divided into 3 sections: hand function (11-items), pain (5-items) and satisfaction (4-items). Each item for hand function is scored from 1 – not difficult to 5 – unable, for level of pain 1 – never to 5 – always and for satisfaction from 1 – very satisfied to 5 – very dissatisfied [19].

#### Data analysis

Descriptive statistics were used to capture the demographics characteristics (age, diagnosis, medications and whether they had surgery or not) of the included sample. A Content Validity Index (CVI) value was computed for each item on the AUSCAN, PRWHE and TDX (I-CVI) as well as for the overall scale (S-CVI). To calculate an item-level CVI (I-CVI), patients with hand arthritis were asked to rate the relevance of each item, on a 4-point scale. Four ordinal points were used foreach scale which was 1 = not relevant, 2 = somewhat relevant, 3 = quite relevant, 4 = highly relevant. Then, for each item, the I-CVI was computed as the number of patients giving a rating of either 3 or 4, divided by the number of raters—that is, the proportion in agreement about relevance and clarity which is between 0 and 1.The S-CVI was calculated by averaging across the I-CVIs of each PROM. To calculate the modified kappa statistic, the probability of chance agreement (Pc) was first calculated for each item by the following formula: Pc = [N! /A! (N -A)!] *0.5^N^ with N being the number of raters (patients with arthritis) and A is the number of patients that agree that the item was clear or relevant [29]. Then Kappa was calculated of entering the probability of chance agreement (Pc) and content validity index of each item (I-CVI) in the following formula: K = (I-CVI - PC) / (1- PC) [29]. Kappa values of 0.74 and above were considered as excellent, 0.60 to 0.74 as good and 0.54 to 0.59 as fair [30]. We performed a Shapiro-Wilk as the omnibus test for assessing univariate normality of each S-CVI distribution, in both relevance and clarity subscales of PROMs. Then, the S-CVI scores were compared with a paired student’s t-Test if normality assumption was met or with Wilcoxon paired signed-ranks test, if assumptions of normality were violated [31]. We conducted all the analyses with STATA (StataCorp. 2019. Stata Statistical Software: Release 16. College Station, TX: StataCorp LLC).

## Results

Overall, 64 individuals with hand arthritis (27% with hand OA, 67% with rheumatoid arthritis in the hand and 6% with psoriatic arthritis) participated in the study. Four individuals were excluded from the analysis because their arthritis was not affecting their hand. The majority of the participants (66%) were taking pain medication on a daily basis (Table 1). All individuals completed the electronic version of the survey.

**Table 1:** Demographics of study participants

Variable	n	Percentage %
**Age, years**		
18–24	1	12%
25–34	8	13%
35–44	13	20%
45–54	17	27%
55–64	19	27%
65–74	5	78%
75–84	1	2%
**Diagnosis**		
Osteoarthritis	17	27%
Rheumatoid arthritis	43	67%
Psoriatic arthritis	4	6%
**Frequency of medication**		
Daily	42	66%
Upon pain	10	16%
Other	12	19%
**Surgery**		
No	49	77%
Yes	15	23%

### Content validity index and modified kappa agreement of the AUSCAN

The I-CVI and the S-CVI supported the content validity of the hand pain, stiffness and function items and subscales of the AUSCANs (Table 2). Five items of pain subscale were rated for relevancy and clarity with I-CVI scores ranging from 0.86 to 0.96 and from 0.92 to 1.00 respectively. For 1-item in stiffness subscale the I-CVI was found 0.93 for relevancy and 1.00 for clarity. For function subscale, 9-items were rated for relevancy and clarity with an I-CVI ranging from 0.88 to 0.97 and from 0.98 to 1.00 respectively. The S-CVI for AUSCAN was found 0.92, 95% CI: 0.90 to 0.94 for relevance and 0.99, 95% CI: 0.98 to 1.00 for clarity. The modified Kappa agreement for every item of the AUSCAN demonstrated excellent agreement (K ranging from 0.86 to 1.00).

**Table 2:** Content validity index of item relevancy and clarity, and Modified Kappa agreement of the Australian and Canadian Osteoarthritis Index (AUSCAN)

Item	Relevance				Clarity				Interpretation
	Agreement	I-CVI^*^	Pc^**^	K^***^	Agreement	I-CVI^*^	Pc^**^	K^***^	
**Rate your pain**									
At rest	49/57	0.86	< 10 ^−5^	0.86	49/50	0.98	< 10 ^− 5^	0.98	Excellent
Gripping	55/57	0.96	< 10 ^− 5^	0.96	49/49	1.00	< 10 ^−5^	1.00	Excellent
Lifting	55/57	0.96	< 10 ^−5^	0.96	49/50	0.98	< 10 ^−5^	0.98	Excellent
Turning	54/57	0.95	< 10 ^−5^	0.95	46/50	0.92	< 10 ^−5^	0.92	Excellent
Squeezing	55/57	0.96	< 10 ^−5^	0.96	50/50	1.00	< 10 ^−5^	1.00	Excellent
**Rate your stiffness**									
After first wakening in the morning	52/56	0.93	< 10 ^−5^	0.93	48/48	1.00	< 10 ^− 5^	1.00	Excellent
**Rate your difficulty when**									
Turning taps/faucets on	51/58	0.88	< 10 ^−5^	0.88	51/51	1.00	< 10 ^−5^	1.00	Excellent
Turning a round doorknob or handle	54/59	0.92	< 10 ^−5^	0.92	53/53	1.00	< 10 ^− 5^	1.00	Excellent
Doing up buttons	52/59	0.88	< 10 ^−5^	0.88	52/52	1.00	< 10 ^−5^	1.00	Excellent
Fastening jewellery	52/59	0.88	< 10 ^− 5^	0.88	53/53	1.00	< 10 ^− 5^	1.00	Excellent
Opening a new jar	57/59	0.97	< 10 ^−5^	0.97	53/53	1.00	< 10 ^− 5^	1.00	Excellent
Carrying a full pot with one hand	56/59	0.95	< 10 ^− 5^	0.95	52/53	0.98	< 10 ^−5^	0.98	Excellent
Peeling vegetables/fruits	56/59	0.95	< 10 ^−5^	0.95	53/53	1.00	< 10 ^−5^	1.00	Excellent
Picking up large heavy objects	55/59	0.93	< 10 ^− 5^	0.93	51/51	1.00	< 10 ^− 5^	1.00	Excellent
Wringing out wash cloths	52/59	0.88	< 10 ^− 5^	0.88	50/51	0.98	< 10 ^−5^	0.98	Excellent
**S - CVI**	**0.92** (95% CI: 0.90 to 0.94)				**0.99** (95% CI: 0.98 to 1.00)				

*NOTE*: *I-CVI: item-level content validity index, **pc (probability of a chance occurrence) was computed using the formula: pc = [N! /A! (N -A)!] *.5^**N**^ where N = number of raters and A = number of raters who agree that the item is relevant or clear, ***K (Modified Kappa) was computed using the formula: K = (I-CVI- PC)/(1- PC). Interpretation criteria for Kappa, using guidelines described in Cicchetti and Sparrow (1981): Fair = K of 0.40 to 0.59; Good = K of 0.60 to 0.74; and Excellent = K > 0.74. I-CVI, item-level content validity index; scale-level content validity index, average (S-CVI/Ave)

### Content validity index and modified kappa agreement of the PRWHE

The I-CVI and the S-CVI of the PRWHE for pain subscale and function subscales all supported the content validity of the PRWHE (Table 3). Five items of pain subscale were rated for relevancy and clarity with I-CVI values ranging from 0.79 to 0.89 and from 0.87 to 0.94, respectively. For function subscales, 10 items were rated for relevancy and clarity with I-CVI values ranging from 0.79 to 0.95 and from 0.92 to 1.00 respectively. The S-CVI for PRWHE was 0.85, 95% confidence intervals (CI): 0.82 to 0.88 for relevance and 0.95, 95%CI: 0.93 to 0.97 for clarity. The modified Kappa agreement for every item of PRWHE demonstrated excellent agreement (K ranging from 0.79 to 1.00).

**Table 3:** Content validity index of item relevancy and clarity and Modified Kappa agreement of Patient Rated Wrist/Hand Evaluation (PRWHE)

Item	Relevance				Clarity				Interpretation
	Agreement	I-CVI^*^	Pc^**^	K^***^	Agreement	I-CVI^*^	Pc^**^	K^***^	
**1. Pain subscale**									
Rate your pain: At rest	51/64	0.80	< 10 ^− 5^	0.80	50/53	0.94	< 10 ^− 5^	0.94	Excellent
Rate your pain: When doing a task with a repeated wrist movement	54/64	0.83	< 10 ^− 5^	0.83	49/53	0.92	< 10 ^− 5^	0.92	Excellent
Rate your pain: When lifting a heavy object	54/64	0.83	< 10 ^−5^	0.83	50/53	0.94	< 10 ^− 5^	0.94	Excellent
Rate your pain: When it is at its worst	57/64	0.89	< 10 ^−5^	0.89	49/53	0.92	< 10 ^−5^	0.92	Excellent
How often do you have pain?	50/63	0.79	< 10 ^−5^	0.79	47/54	0.87	< 10 ^−5^	0.87	Excellent
**2. Function**									
**A. Specific activities**									
Turn a doorknob using my affected hand	53/63	0.84	< 10 ^−5^	0.84	52/52	1.00	< 10 ^−5^	1.00	Excellent
Cut meat using a knife in my affected hand	54/63	0.86	< 10 ^−5^	0.86	53/53	1.00	< 10 ^− 5^	1.00	Excellent
Fasten buttons on my shirt	51/63	0.81	< 10 ^−5^	0.81	53/53	1.00	< 10 ^−5^	1.00	Excellent
Use my affected hand to push up from a chair	50/63	0.79	< 10 ^− 5^	0.79	51/52	0.98	< 10 ^−5^	0.98	Excellent
Carry a 10 lb. object in my affected hand	58/63	0.92	< 10 ^−5^	0.92	52/53	0.98	< 10 ^−5^	0.98	Excellent
Use bathroom tissue with my affected hand	50/63	0.79	< 10 ^−5^	0.79	51/52	0.98	< 10 ^− 5^	0.98	Excellent
**B. Usual activities**									
Personal care activities (dressing, washing)	53/61	0.87	< 10 ^− 5^	0.87	50/53	0.94	< 10 ^−5^	0.94	Excellent
Household work (cleaning, maintenance)	57/60	0.95	< 10 ^−5^	0.95	49/53	0.92	< 10 ^−5^	0.92	Excellent
Work (your job or usual everyday work)	52/60	0.87	< 10 ^− 5^	0.87	49/53	0.92	< 10 ^− 5^	0.92	Excellent
Recreational activities	54/60	0.90	< 10 ^− 5^	0.90	51/53	0.96	< 10 ^− 5^	0.96	Excellent
**S – CVI/Ave**	**0.85** (95% CI: 0.82 to 0.88)				**0.95** (95% CI: 0.93 to 0.97)				

*NOTE*: *I-CVI: item-level content validity index, **pc (probability of a chance occurrence) was computed using the formula: pc = [N! /A! (N -A)!] *.5^**N**^ where N = number of raters and A = number of raters who agree that the item is relevant or clear, ***K (Modified Kappa) was computed using the formula: K = (I-CVI- PC)/(1- PC). Interpretation criteria for Kappa, using guidelines described in Cicchetti and Sparrow (1981): Fair = K of 0.40 to 0.59; Good = K of 0.60 to 0.74; and Excellent = K > 0.74. I-CVI, item-level content validity index; scale-level content validity index, average (S-CVI/Ave)

### Content validity index and modified kappa agreement of the TDX

The I-CVI and the S-CVI supported the content validity of the TDX for hand function, pain and satisfaction subscales (Table 4). Eleven items of hand function were rated as relevant and clear with I-CVI values ranging from 0.82 to 0.93 and from 0.94 to 0.98 respectively. For pain subscale, five items were rated as relevant and clarity with I-CVI scores ranging from 0.78 to 0.85 and from 0.77 to 0.86 respectively. For the satisfaction subscale, four items were rated as relevant and clear based on I-CVI demonstrating scores from 0.83 to 0.95 and from 0.88 to 0.91. The S-CVI of TDX was rated as relevant and clear based on scores of 0.87, 95% CI: 0.85 to 0.89 for relevancy and 0.91, 95% CI: 0.89 to 0.94 for clarity. The modified Kappa agreement demonstrated excellent inter-rater agreement on item ratings (K ranging from 0.77 to 0.98).

**Table 4:** Content validity index of item relevancy and clarity, and Modified Kappa agreement of the Thumb Disability Exam (TDX)

Item	Relevance				Clarity				Interpretation
	Agreement	I-CVI^*^	Pc^**^	K^***^	Agreement	I-CVI^*^	Pc^**^	K^***^	
**A. Please indicate your ability to perform these activities with the affected hand**									
Turn a Key	54/61	0.89	< 10 ^− 5^	0.89	51/53	0.96	< 10 ^− 5^	0.96	Excellent
Pick up a coin	52/61	0.85	< 10 ^− 5^	0.85	49/51	0.96	< 10 ^− 5^	0.96	Excellent
Write	56/61	0.92	< 10 ^− 5^	0.92	51/54	0.94	< 10 ^−5^	0.94	Excellent
Squeeze toothpaste	52/60	0.87	< 10 ^−5^	0.87	51/53	0.96	< 10 ^−5^	0.96	Excellent
Hold a glass of water	50/61	0.82	< 10 ^− 5^	0.82	51/54	0.94	< 10 ^− 5^	0.94	Excellent
Turn a doorknob	52/61	0.85	< 10 ^− 5^	0.85	51/53	0.96	< 10 ^− 5^	0.96	
Use a knife to cut food	54/61	0.89	< 10 ^−5^	0.89	51/53	0.96	< 10 ^−5^	0.96	Excellent
**B. Please indicate your ability to perform the following task while using both your hands**									
Open a jar	57/61	0.93	< 10 ^−5^	0.93	50/51	0.98	< 10 ^−5^	0.98	Excellent
Button a shirt/blouse	53/61	0.87	< 10 ^−5^	0.87	49/50	0.98	< 10 ^−5^	0.98	Excellent
Tie your shoes	55/61	0.90	< 10 ^−5^	0.90	50/51	0.98	< 10 ^−5^	0.98	Excellent
Wring a dishcloth/washcloth	53/61	0.87	< 10 ^−5^	0.87	49/51	0.96	< 10 ^−5^	0.96	Excellent
**II. The following questions refer to the level of pain in your thumb**									
How often did you have pain in your thumb at rest?	50/61	0.82	< 10 ^−5^	0.82	40/52	0.77	< 10 ^−5^	0.77	Excellent
How often did the pain in your thumb interfere with your daily activities?	49/60	0.82	< 10 ^−5^	0.82	44/51	0.86	< 10 ^−5^	0.86	Excellent
How often did the pain in your hand interfere with recreational activities?	51/60	0.85	< 10 ^−5^	0.85	44/52	0.85	< 10 ^−5^	0.85	Excellent
How often did the pain in your thumb interfere with your sleep?	47/60	0.78	< 10 ^−5^	0.78	44/52	0.85	< 10 ^−5^	0.85	Excellent
How often did the pain in your thumb worsen your mood?	51/60	0.85	< 10 ^−5^	0.85	42/52	0.81	< 10 ^−5^	0.81	Excellent
**III. The following questions ask about your satisfaction with the indicated hand or thumb over the past week.**									
Motion in your affected thumb	48/58	0.83	< 10 ^−5^	0.83	47/53	0.89	< 10 ^−5^	0.89	Excellent
Strength of your affected hand	54/57	0.95	< 10 ^−5^	0.95	48/53	0.91	< 10 ^−5^	0.91	Excellent
Pain level of your affected hand	52/58	0.90	< 10 ^−5^	0.90	48/53	0.91	< 10 ^−5^	0.91	Excellent
Overall function of your hand	53/58	0.91	< 10 ^−5^	0.91	46/52	0.88	< 10 ^−5^	0.88	Excellent
**S-CVI**	**0.87** (95% CI: 0.85to 0.89)				**0.91** (95% CI: 0.89to 0.94)				

*NOTE*: *I-CVI: item-level content validity index, **pc (probability of a chance occurrence) was computed using the formula: pc = [N! /A! (N -A)!] *.5^**N**^ where N = number of raters and A = number of raters who agree that the item is relevant or clear, ***K (Modified Kappa) was computed using the formula: K = (I-CVI- PC)/(1- PC). Interpretation criteria for Kappa, using guidelines described in Cicchetti and Sparrow (1981): Fair = K of 0.40 to 0.59; Good = K of 0.60 to 0.74; and Excellent = K > 0.74. I-CVI, item-level content validity index; scale-level content validity index, average (S-CVI/Ave)

## Discussion

This study established a high level of content validity for AUSCAN, PRWHE and TDX for patients with hand arthritis. The content validity index was very high for all the individual items for each questionnaire (I-CVI > 0.77) and for the overall score (S-CVI > 0.85) in terms of relevancy and clarity, exceeding the recommended benchmarks of 0.78 respectively [29]. The Kappa inter-rater agreement of > 0.75 was excellent across all the individual items for all PROMs (AUSCAN, PRWHE and TDX) among the raters [29]. Together these data provide confidence in our assessment since multiple raters agreed on the high content validity scores obtained.

For the AUSCAN the content validity was established during development using a formal clinimetric process where patients in a tertiary care centre rated items by importance and frequency to establish relevance [20]. This study provides additional support for the content validity in a community sample of people living with hand arthritis, and by adding new data on the clarity of the items.

Content validity of PRWHE was established during the development of the PRWHE by using semi-structured interviews in patients with distal radius fracture and expert opinion [32]. Later the extension to the PRWHE compared relevance to DASH, based on a comparative trial in a mixed clinical population with hand problems. However, neither were quantified, described specific findings in-depth or focused on patients with arthritis. Thus, this study provides novel information on the content validity of the items of the PRWHE, with specific reference to those with hand arthritis. All items of PRWHE were found with very high content validity index in terms of relevance (I-CVI > 0.79) and clarity (I-CVI > 0.87).

It might have been expected that the AUSCAN would have more relevance to our sample, than the PRWHE since it a disease-specific PROM. Both point estimate and CI comparisons indicate that AUSCAN had slightly higher overall scores in terms of relevancy (S-CVI = 0.92, 95% CI: 0.90 to 0.94) and clarity (S-CVI = 0.99, 95% CI: 0.98 to 1.00) than the PRWHE (S-CVI = 0.85, 95% CI: 0.82 to 0.88 for relevancy and S-CVI = 0.95, 95% CI: 0.93 to 0.97 for clarity). Although the CIs of the respective S-CVIs indicate that there was a small statistically significant difference (Table 5) between compared S-CVI values (AUSCAN vs TDX and AUSCAN vs PRWHE), all PROMs met standards of very high content validity. Further, since 6 to 8 additional raters assessed the PRWHE that did not assess the AUSCAN, the small differences may reflect differences in rater pools rather than an actual difference in perceptions.

**Table 5:** Comparison of content validity index (S-CVI) of relevance and clarity

Relevance				Clarity			
	PRWHE	TDX	AUSCAN		PRWHE	TDX	AUSCAN
PRWHE	0.85 (95% CI: 0.82–0.88)	*Paired* *t-Test*	*Paired* *t-Test*	PRWHE	0.95 (95% CI: 0.93–0.97)	*Wilcoxon Signed ranks*	*Wilcoxon Signed ranks*
TDX	*p* = 0.523	0.87 (95% CI: 0.85–0.89)	*Paired* *t-Test*	TDX	*p* = 0.153	0.91 (95% CI: 0.89–0.94)	*Wilcoxon Signed ranks*
AUSCAN	*p* < 0.001	*p* = 0.001	0.92 (95% CI: 0.90–0.94)	AUSCAN	*p* = 0.001	*p* = 0.002	0.99 (95% CI: 0.98–1.00)

Paired t-Test: Student’s t-Test for Matched Pairs; Wilcoxon Signed Ranks: Wilcoxon Matched-Pairs Signed-Ranks

*AUSCAN* Australian and Canadian Osteoarthritis Index, *TDX* Thumb Disability Exam, *PRWHE* Patient Rated Wrist/Hand Evaluation

The TDX is relatively new developed PROM (Noback et al. 2017) [19] that was tested in patients with basal joint arthritis. The TDX demonstrated very high content validity index when assessed in terms of relevancy (S-CVI = 0.87, 95% CI: 0.85 to 0.89) and clarity (S-CVI = 0.91, 95% CI: 0.89 to 0.94). All the individual items of the TDX had a very high content validity index (I-CVI > 0.77). No previous studies have reported the content validity index of TDX. The item generation of TDX included the review of items from relevant scales (Michigan Hand Questionnaire (MHQ) [33], Disabilities of the Arm, Shoulder, and Hand (DASH) [34], AUSCAN [20], PRWHE [27] and McGill Pain questionnaire [35]). Then, the development process included item reduction and pilot testing and then final item reduction [19]. Thus the items may have benefited from content validity efforts made in developing the scales. Since the thumb is so important for overall hand function, it is not surprising that this thumb questionnaire was found to have validity for patients with hand arthritis.

Our kappa statistics indicated excellent agreement between patient raters after correcting for chance agreement. (K > 0.77). The assessment from a large pool of patients (*n* > 60) generated similar scores between the I-CVI and K scores. This has been previously described in the literature when the number of raters increasing and the probability of chance (Pc) decreases the K agreement and I-CVI values tend to converge [29].

This study provided novel data on the content validity index in 3 different PROMs in patients with hand arthritis. Since few studies address content validity, this is important to support the conceptual foundations of these measures and support their use in clinical practice. While the computation of CVI is relatively easy, its major weakness is the failure to adjust for chance agreement. However, the authors tried to mitigate this problem by calculating a modified kappa agreement [29, 36]. A potential limitation is that the items of the PROMs were not randomized but the items were rated for relevance and clarity in an order (PRWHE, AUSCAN, TDX). Since all three scales were brief, we would think it is unlikely that there was an order effect, especially since the highest scores were found in the questionnaire administered in the middle. CVI is one method of assessing content validity and as a quantitative process are ideally suited to rating existing items, not to identification of potential gaps in important constructs. Ideally CVI should be augmented by qualitative techniques like cognitive interviewing or understanding the dimensions of the underlying construct to be measured. Also, all three questionnaires demonstrated high content validity, and existing evidence confirms that all three provide strong psychometric properties then practical considerations might be the predominant difference that would guide selection. For example, the AUSCAN requires that a license fee must be paid to the developer, whereas the other questionnaires are copyrighted but freely available for all users.

## Conclusions

This study demonstrated evidence of very high content validity index for all the individual items and for the overall scale of AUSCAN, PRWHE and TDX for patients with hand arthritis, with high agreement across raters. This augments prior statistical evidence supporting statistical measurement properties, to provide support for the content validity.
